# Time-Shifted Maps for Industrial Data Analysis: Monitoring Production Processes and Predicting Undesirable Situations

**DOI:** 10.3390/s25113311

**Published:** 2025-05-24

**Authors:** Tomasz Blachowicz, Sara Bysko, Szymon Bysko, Alina Domanowska, Jacek Wylezek, Zbigniew Sokol

**Affiliations:** 1 PROPOINT S.A., R&D Department, Bojkowska 37 R Str., 44-100 Gliwice, Poland; szymon.bysko@propoint.pl (S.B.); jacek.wylezek@propoint.pl (J.W.); zbigniew.sokol@propoint.pl (Z.S.); 2 Institute of Physics—CSE, Silesian University of Technology, S. Konarskiego 22B Str., 44-100 Gliwice, Poland; alina.domanowska@polsl.pl; 3 Faculty of Automatic Control, Electronics and Computer Sciense, Silesian University of Technology, Akademicka 16 Str., 44-100 Gliwice, Poland; sara.bysko@polsl.pl

**Keywords:** industrial data analysis, monitoring of industrial processes, predictive maintenance, production in a robotic cell

## Abstract

The rapid advancement of computing power, combined with the ability to collect vast amounts of data, has unlocked new possibilities for industrial applications. While traditional time–domain industrial signals generally do not allow for direct stability assessment or the detection of abnormal situations, alternative representations can reveal hidden patterns. This paper introduces time-shifted maps (TSMs) as a novel method for analyzing industrial data—an approach that is not yet widely adopted in the field. Unlike contemporary machine learning techniques, TSM relies on a simple and interpretable algorithm designed to process data from standard industrial automation systems. By creating clear, visual representations, TSM facilitates the monitoring and control of production process. Specifically, TSMs are constructed from time series data collected by an acceleration sensor mounted on a robot base. To evaluate the effectiveness of TSM, its results are compared with those obtained using classical signal processing methods, such as the fast Fourier transform (FFT) and wavelet transform. Additionally, TSMs are classified using computed correlation dimensions and entropy measures. To further validate the method, we numerically simulate three distinct anomalous scenarios and present their corresponding TSM-based graphical representations.

## 1. Introduction

The rapid development of big data processing technologies has revolutionized various industries by enabling the collection, storage, and analysis of vast amounts of data. This transformation is particularly evident in industrial settings, where data-driven approaches are increasingly employed to optimize processes, enhance efficiency, and ensure product quality. The integration of artificial intelligence (AI) and machine learning (ML) with big data has opened new ways for predictive analytics, anomaly detection, and process optimization, marking a shift toward smarter and more adaptable industrial systems [1,2,3,4,5,6]. However, leveraging big data in industrial contexts is not without challenges. The complexity arises from the dynamic nature of manufacturing systems, variability in operational conditions, and the need to balance efficiency, quality, and cost [7]. Moreover, industrial data often originate from heterogeneous sources, such as sensors, logs, and machine controllers, and are characterized by high dimensionality, noise, and variability [8,9]. An essential aspect of industrial data analysis is anomaly detection, which plays a critical role in maintaining system reliability and production continuity. Various methods, including supervised and unsupervised machine learning, have been applied to detect anomalies in complex systems [10,11,12,13,14]. Despite their effectiveness, these methods often require significant computational resources, specialized expertise, and large labeled datasets, which can limit their accessibility for small and medium-sized enterprises (SMEs) [15,16,17]. However, this remains a complex challenge, depending on factor such as data scalability, the feasibility of real-time processing, access to predictive analytics, the type of industrial sector, and the methods of data visualization [18,19,20,21,22,23,24,25,26,27,28,29,30,31].

Industrial data processing is inextricably linked to signal analysis with numerous well-established methods and techniques available in the field. Specifically, because industrial processes typically exhibit repeatable characteristics due to serial production and repetitive operations, methods such as the FFT [32,33,34] and wavelet analysis [35,36,37,38,39] are commonly used to extract frequency–domain and time–frequency characteristics, respectively. Well-known statistical analysis methods, such as the Gaussian distribution [40,41], also play a key role, providing fundamental insights into measures like standard deviation. Another less commonly known method for the statistical analysis of rare or anomalous events is the use of copula functions [42].

In this study, we propose a new approach and evaluate it under realistic industrial conditions. Specifically, we analyze data from an acceleration sensor mounted on the mechanical base of a robot, using time-shifted mapping (TSM). A similar technique, often applied in deterministic chaos studies [43,44], explores the relationships between position and velocity or velocity and acceleration to identify periodicities or anomalies in nonlinear physical system dynamics. However, the TSM approach is used to detect periodicity and repeatability in the robot’s operation within its cell. Additionally, by superimposing artificial perturbations on the signals, we examined the following anomalies: increasing random fluctuations, linear decay, and a rapid, temporary decrease in amplitude (a “time well”). These anomalies mimic scenarios such as increased random vibration due to loosening or broken connections in the robot cell, the gradual loss of actuator movement from a power supply drop, or abrupt movement interruptions caused by mechanical or power failures. This paper provides details on the data origin, introduces the TSM approach, analyzes the data, and presents final remarks. The goal is to propose a simple, user-friendly graphical method for industrial data analysis to enhance production quality.

To enhance the quantitative interpretation of TSM graphical results, we propose two numerical descriptors of TSM images. First, we use correlation dimension analysis, and second, metric entropy calculated from the image [45]. Additionally, to enhance the interpretability of our proposed method, we also implement FFT analysis and the continuous wavelet transform (CWT) using Morlet and Ricker wavelets.

The paper is organized as follows. The next section presents the theoretical foundations of the TSM approach, including results related to deterministic chaos in mechanical systems. The second part of this section provides details on the preparation of TSM graphics. The next section presents experimental results obtained from an acceleration sensor mounted on the working base of a robot. The results are analyzed using well-established methods, namely the FFT and CWT. Notably, the FFT approach proved particularly helpful for interpreting the so-called time shift of data—a fundamental step in the TSM method. At the end of this chapter, we present the numerical characteristics of the maps, namely the correlation dimensions and entropies, calculated for all four aforementioned cases. In this section, it will be demonstrated that the TSM method visualizes signal changes in a visually direct and quantitative manner, showing its advantage over the FFT and CWT methods. This article concludes with a summary and final conclusions.

## 2. Theoretical Foundations

### 2.1. Deterministic Chaos as Example of Complex Behavior of Mechanical Systems

In many fields of science and technology, particularly in dynamic systems where operating parameters vary over time, it is possible to analyze a selected parameter using appropriate spectral analysis methods. Among the wide variety of signals, two extreme cases can be distinguished: signals with well-defined periodicity, for which classical Fourier analysis is highly effective, and completely aperiodic, random signals, for which wavelet analysis can be a suitable method.

The periodicity of the signal x(t) can be expressed as:

(1) x(t)=x(t+T)

where T is the time period. For signals exhibiting multiple frequencies simultaneously, ($f_{1},\;f_{2},\ldots ,\;f_{n})$, additional secondary frequencies appear, and the resulting complex signal spectrum can depend on the amplitude of the individual components. For example, for signals with two primary frequencies, $f_{1}$ and $f_{2}$, the resulting signal may include frequencies corresponding to their sum and difference, i.e., $f_{1}-f_{2}$ and $f_{1}+f_{2},\;$ respectively. Additionally, higher-order frequency components such as ${2f}_{1}-f_{2}$, ${2f}_{1}+f_{2}$, ${2f}_{1}-2f_{2}$, ${2f}_{1}+2f_{2}$, and other multiples of sums and differences may also appear, though usually with vanishingly small amplitudes.

A crucial issue in industrial signals, which has practical significance, is whether periodicity is synonymous with defect occurrence. This question is directly related to the concept of deterministic chaos, i.e., the possibility of a physical system predictably transitioning into aperiodic behavior.

For dynamical systems governed by deterministic chaos, several well-established analytical methods are used, often with convenient graphical representations of system variability. These include phase diagrams, Poincaré sections, and bifurcation diagrams. In this work, we do not strictly apply deterministic chaos methods but instead focus on the analysis of two-dimensional phase diagrams applied to time-varying acceleration signals measured at selected points in a robotic cell, e.g., using a sensor attached to the base of a welding robot.

**Figure 1:**
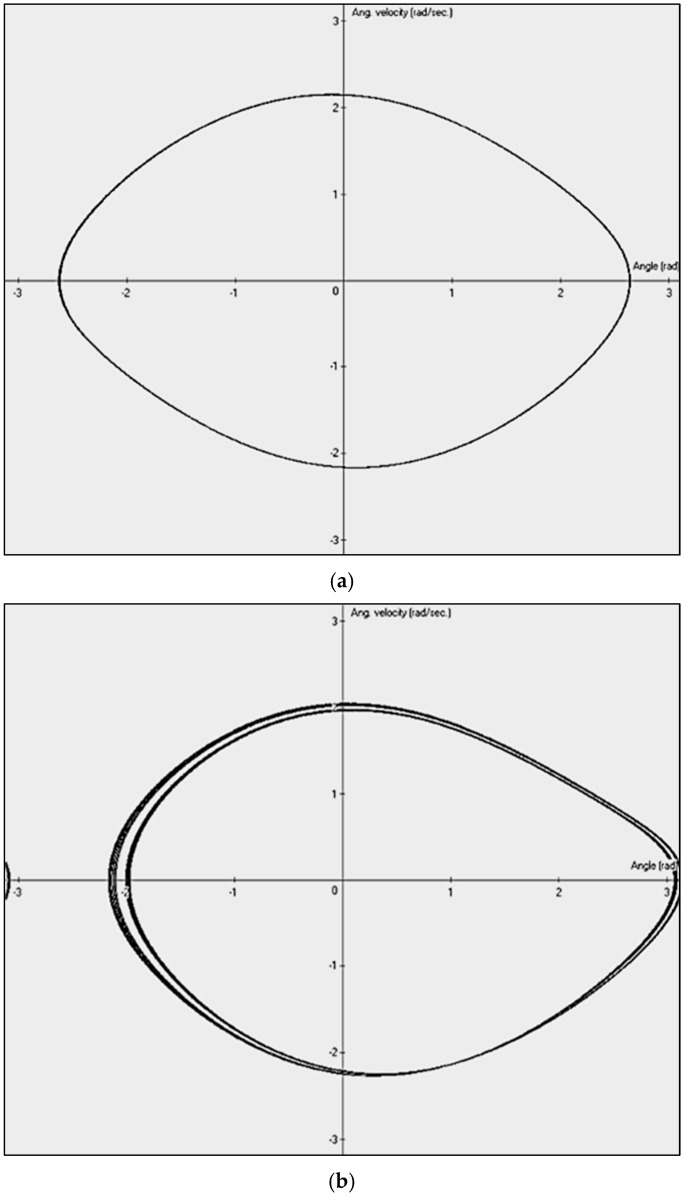

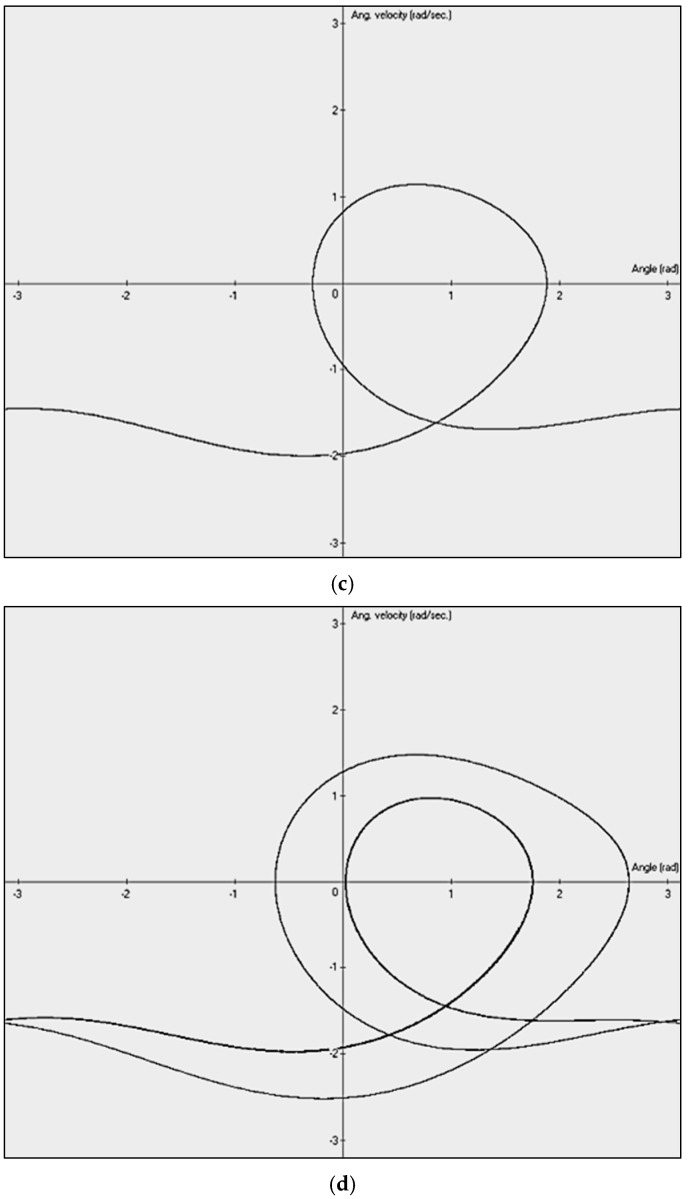

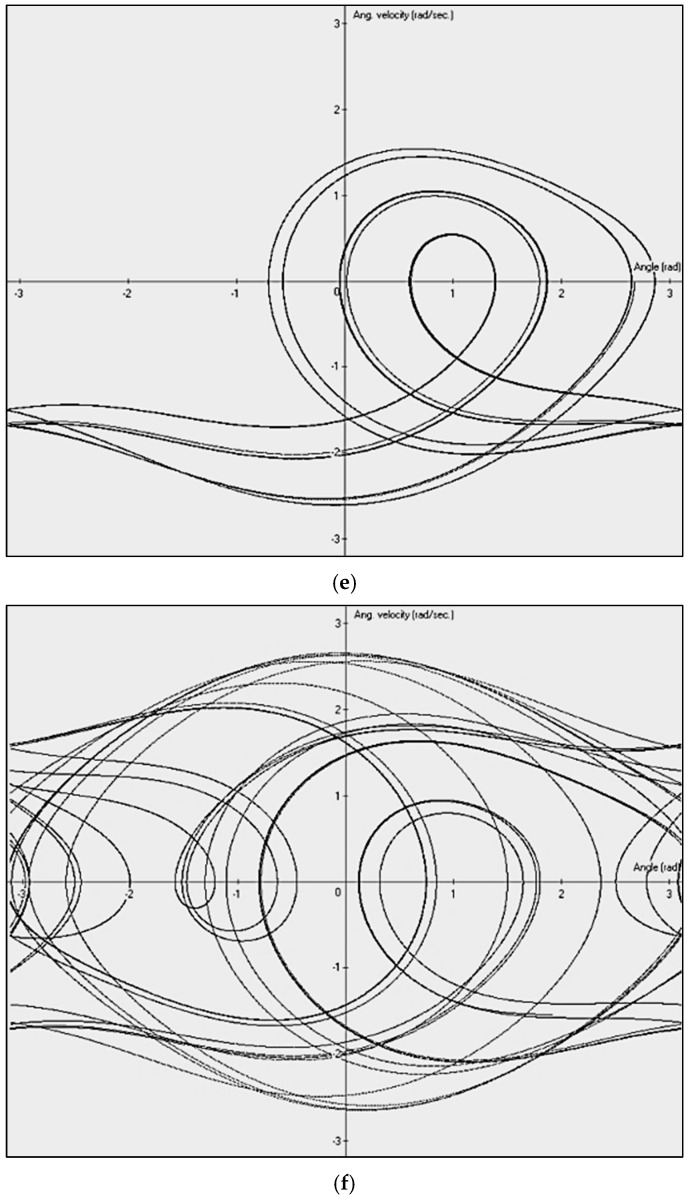
Phase diagrams of damped driven physical pendulum for different driving moments of force amplitude C′ (Equation (6)): single frequency case, $C^{\prime }=1.0$ (**a**), doubled frequency case, $C^{\prime }=1.07$ (**b**), single frequency case, for larger amplitude $C^{\prime }=1.35$ (**c**), doubled frequency case, $C^{\prime }=1.45$ (**d**), quasi-chaotic state, $C^{\prime }=1.47$ (**e**), and fully chaotic state, $C^{\prime }=1.50$ (**f**).

A phase diagram, in its basic form, represents the relationship between the rate of change of a time-varying signal dx/dt and the signal itself, x(t). For a perfect sinusoidal signal $x\left(t\right)=Asin(\omega t)$, its first time derivative is $x^{\prime }(t)=A\omega cos\left(\omega t\right)=Bcos\left(\omega t\right)$. The phase diagram, i.e., the plot of $x^{\prime }\left(t\right)$ as a function of x(t), can form an elliptical shape (comp. Figure 1a. In the following section, we will show that the phase diagram ($x^{\prime }\left(t\right)$ vs. $x\left(t\right)$) is equivalent to the diagram ($x\left(t+\tau \right)$ vs. $x\left(t\right)$), where τ is the time shift. This concept is named time-shifted mapping (TSM) of signals. By selecting an appropriate time shift, it is possible to distinguish between normal and abnormal conditions. In the actual paper, the analysis was performed using real process signals recorded from a working welding robot in a robotic cell. Failure scenarios were simulated to represent extreme conditions.

From a practical perspective in an industrial setting, real signals are often perturbed and can be represented as:

(2) $$x(t)=A\left(t\right)sin(\omega t+\delta (t))$$

where, in general, the phase disturbance function δ(t) can be periodic, aperiodic, or even random. Similarly, the signal amplitude $A\left(t\right)$ may undergo random or, less frequently, periodic variations. For an idealized process, it can be assumed $\delta \left(t\right)=0$ and $A\left(t\right)=const$.

In a typical industrial process, such as cyclically repeated welding in serial production, periodic variations in technological parameters naturally occur. A potential monitoring parameter, treated as a time-varying signal following the production cycle, could be the acceleration measured at various locations in the robotic cell by a point sensor.

Returning to the example of simple sinusoidal signals, x=Asin(ωt) and y=Bcos(ωt), the problem of graphical representation of periodicity reduces to finding the relation y=y(x). Eliminating the explicit time dependence leads to squaring the signals and using the trigonometric identity ${sin}^{2}\left(\omega t\right)+{cos}^{2}\left(\omega t\right)=1$, yielding:

(3) $${\left(\frac{x}{A}\right)}^{2}+{\left(\frac{y}{B}\right)}^{2}=1.$$

Similarly, for a perturbed signal of the form $x(t)=A\left(t\right)sin(\omega t+\delta (t))$, we have:

(4) $${\left(\frac{x}{A(t)}\right)}^{2}+{\left(\frac{y}{B(t)}\right)}^{2}=1,$$

However, experimental results in the field of deterministic chaos show that the topology of phase diagrams can differ significantly from those generated by simple trigonometric functions. The analysis presented in this paper partially overlaps with the studies of deterministic chaos, which can have practical applications in the analysis of real mechanical systems. As a concrete example of a system with more complex behavior than a simple harmonic oscillator (Equation (3)), we present phase diagrams for a driven, damped physical pendulum. This approach is discussed in detail in Baker and Golub’s book [31], based on which we developed a simple numerical simulator to illustrate these results. The equation of motion for the pendulum is given by:

(5) $$-Asin\left(\alpha \right)-B\frac{d\alpha }{dt}+Ccos\left(\omega t\right)=D\frac{d^{2}\alpha }{dt^{2}},$$

where A is gravitation factor, B is the damping factor, C is the amplitude of the externally applied moment of force, which is applied periodically with the frequency ω, and D is the moment of inertia. The equation in its normalized form ($A/D=1,B/D=B^{\prime },C/D=C^{\prime }$) can be written as follows:

(6) $$-sin\left(\alpha \right)-B^{\prime }\frac{d\alpha }{dt}+C^{\prime }cos\left(\omega t\right)=\frac{d^{2}\alpha }{dt^{2}}.$$

In the following, we will refer to different values of the C’ amplitude (1.0, 1.07, 1.35, 1.45, 1.47, 1.50) to illustrate some results, assuming ω=2/3 (rad/sec.) and $B^{\prime }=0.5$. Thus, the behavior of the pendulum is governed by three key parameters (as a minimum of three parameters is required for chaotic behavior): the damping coefficient $B^{\prime }$, the excitation frequency ω of the external driving force, and the amplitude of the external driving force $C^{\prime }$. By selecting appropriate parameter values, different dynamic behaviors can be observed. These include cyclic motion with a single dominant frequency (Figure 1a,c)—visible in the phase diagram as a single oval, topologically similar to a perfect ellipse (Figure 1a)—as well as more complex cases, such as period-doubling bifurcations (two-cycle behavior) (Figure 1b,d), the quasi-chaotic state (Figure 1e), or the fully chaotic case (Figure 1f).

The focus of this article is the presentation of such diagrams, derived from real industrial data. For visualization, we employ time-shifted diagrams, where a time-shifted signal is used to approximate the rate of change of the original signal. The key conclusion drawn from these findings is that in dynamic systems influenced by multiple factors (parameters), phase diagrams can exhibit highly complex topologies.

### 2.2. The Concept of Time-Shifted Mapping

**Figure 2:**
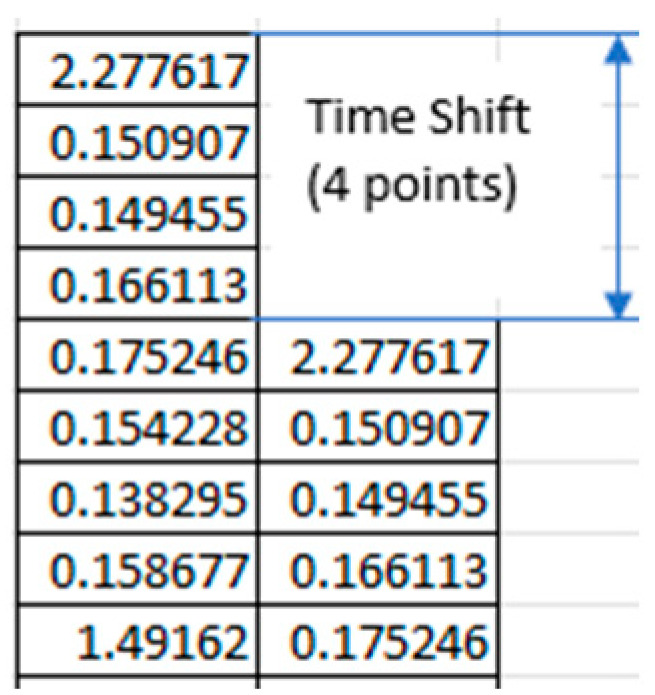
Basic data manipulation in the concept of time-shifted maps. A one-dimensional signal (a column of data) is duplicated and shifted downward by a specified number of data cells. This time shift—denoted by T in the following discussion—is referred to as a “point” in terms of the number of discrete data cells (in this example, it equals 4).

**Figure 3:**
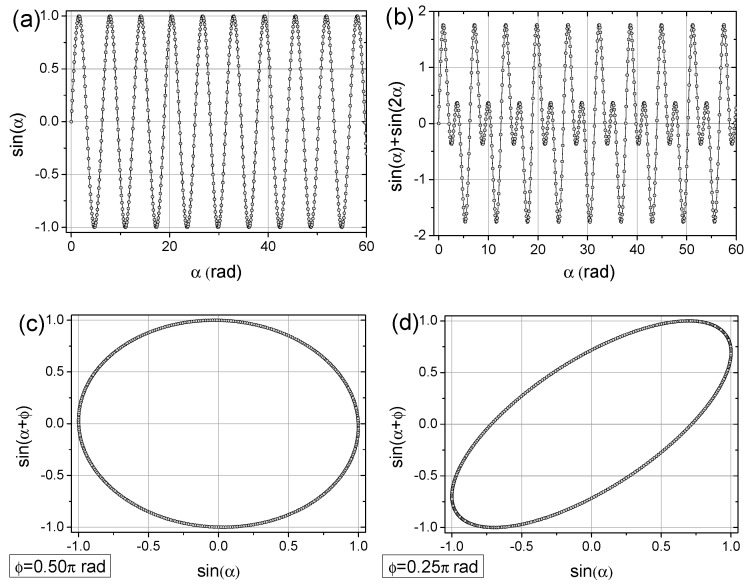

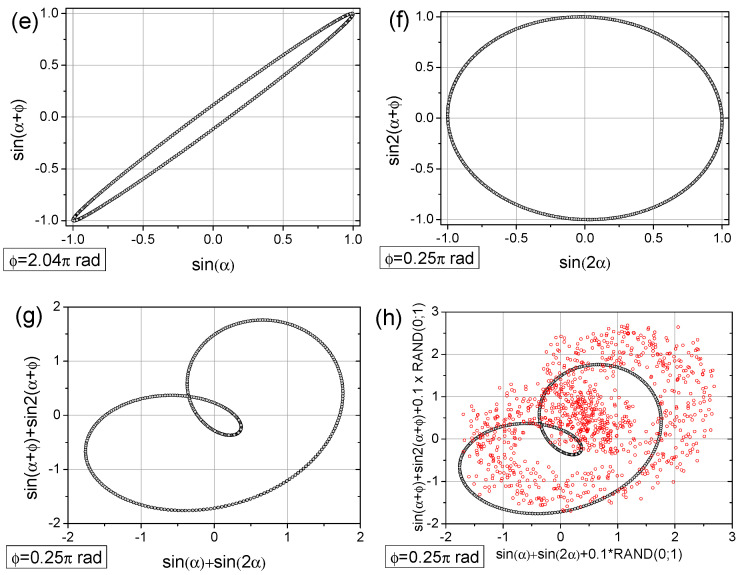
The concept of time-shifted maps for examining periodicity in industrial signals. Numerical values were calculated with a resolution of 0.1 rad. (**a**) The basic sin(α) data series; (**b**) the two-fold more frequent signal sin(2α); (**c**) the sin(α + φ) vs. sin(α) dependence with φ = 0.50π rad; (**d**) the sin(α + φ) vs. sin(α) dependence with φ = 0.25π rad; (**e**) the sin(α + φ) vs. sin(α) dependence with φ = 2.04π rad; (**f**) the sin[2(α + φ)] vs. sin(2α) dependence with φ = 0.25π rad; (**g**) the sin(2α) + sin[2(α + φ)] vs. sin(α) + sin(2α) dependence with φ = 0.25π rad; and (**h**) the sin(2α) + sin[2(α + φ)] vs. sin(α) + sin(2α) dependence, with φ = 0.25π rad along the imposed random noise (red points).

The basic step in realizing TSM, that is, shifting a copied data series relative to the original series, is shown in Figure 2. To understand more deeply the concept of time-shifted maps, let us consider two simple sinusoidal functions: a single sin⁡(α) function (Figure 3a) with a 2π period, and the sum of sin⁡(α) and the two-fold more frequent sin⁡(2α) function. In the next step, we can prepare the sin⁡(α+ϕ) vs. sin⁡(α) plot, where ϕ represents the phase shift. By comparing Figure 3c–e, with phase shift of ϕ=0.50π, ϕ=0.25π, ϕ≈2.0π, respectively, we observe that for a phase shift equal to ¼ of the period, the graphical representation in the form of single oval indicates a single-period signal (Figure 3c). Similarly, for the function with double the frequency, the ϕ=0.25π shift clearly reveals a single-period signal with twice the frequency (Figure 3f). Next, by applying the same approach with ϕ=0.25π to the combination $\sin\left(\alpha \right)+sin(2\alpha )$, we obtain a map with two loops, indicating two types of periodicities (Figure 3g). Furthermore, by superimposing random values (noise) onto the two-cycled signal, the periodicities of the pure signals can still be recognized (Figure 3f).

These simple examples provide, of course, only an approximation of industrial situations. Nevertheless, as will be shown below, analyzing time-collected acceleration data offers new insights into the nature of signals. Thus, it is easy to envision scenarios where the periodic nature of the data is either unknown or not immediately apparent, especially when the collected time series are not represented by simple trigonometric functions. As will be demonstrated below, time-shifted maps exhibit a significant resolution capability across various cases, including unpredictable and undesirable ones.

As it was mentioned above, in chaotic deterministic systems, analyzing the relationships between dynamical variables (e.g., generalized positions) and their velocities is a common practice [26]. The relationships can be presented as two-dimensional figures. From a numerical perspective, in the simplest case, the relationship between position $x_{i+1}$ at a later time and the position at an earlier time $x_{i}$ can be expressed as

(7) $$\frac{x_{i+1}-x_{i}}{∆t}=Ax_{i}$$

where ∆t is the time step value, and A is the proportionality factor. After some straightforward derivations, it can be shown that

(8) $$x_{i+1}=\left(A∆t+1\right)x_{i}=C\left(t\right)x_{i}$$

with a time-dependent proportionality factor $C\left(t\right)$, this means the velocity–position diagram (related to Equation (7)) can be used interchangeably with appropriately time-shifted position data (related Equation (8)) to obtain an adequate graphical representation of the dynamics of the system under analysis. It is worth noting that the maps can be created using different values of the time shift ∆t.

## 3. Experiment and Analysis of Data

The automation system developed for the experiment was divided into three interconnected layers: control, visualization with HMI (human–machine interface), and SCADA (supervisory control and data acquisition). The main component of the control layer was the PLC, which managed the operation of all actuators and measuring devices, enabling the process to run in automatic mode. The Siemens S7-1500 controller (Siemens, Munich, Germany) used in the experiment had been programmed in the TIA Portal environment. The visualization layer consisted of a screen and touch panel integrated with an industrial PC. Visualization and control of the system were implemented using Zenon 8.2 software from COPA-DATA (Salzburg, Austria). This software employed a communication plug-in mechanism to connect with Siemens controllers in various ways, with a focus on the OPC-UA (Open Platform Communication—Unified Architecture) communication protocol, which was one of the most promising technologies within the Industry 4.0 framework. The SCADA system fulfilled several roles. Primarily, it acted as a central hub for data collected from the control layer. This data were analyzed for diagnostics, optimization, failure prediction, and production status reporting. The analysis results were displayed within the HMI layer. Given the cell’s ability to function in both stand-alone and collaborative modes, the SCADA system had been divided into two subsystems: a local subsystem tied to the PLC and a global subsystem supporting the operation of multiple robot cells. The current experiment utilized the local subsystem.

**Figure 4:**
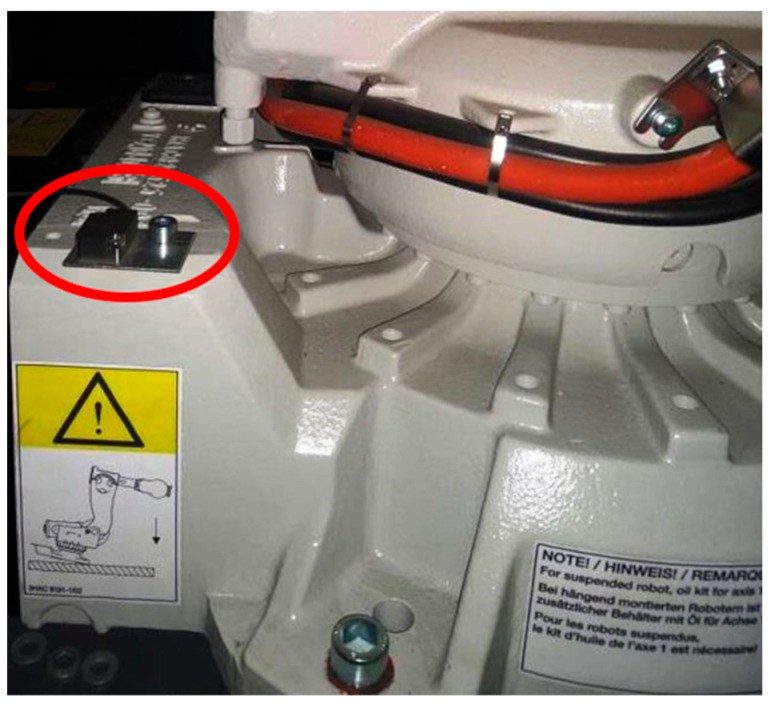
An acceleration sensor (highlighted by the red oval) is mounted on the base of the robot.

The data were collected using an acceleration sensor (Baluff BCM0001, Neuhausen auf den Fildern, Germany) [46] mounted on the mechanical base of the robot (Figure 4). The Kuka KR 270 robot [47] (Augsburg, Germany) was installed inside a fully enclosed robotic cell measuring approximately 3 m × 3 m × 3 m. The walls of the cell consisted of uniform, flat steel sheets mounted on a frame and connected to a ground frame made of T-bars, which also supported the robot base. All components of the cell enclosure were securely fastened with bolted or welded joints. This setup ensured that vibrations generated by the robot propagated throughout the structure, particularly to the robot’s base and walls of the robotic cell.

The robot performed repetitive movements related to the welding process of small metallic parts secured to a rotary table. The entire experiment lasted 2700 s, with acceleration and velocity measured at 0.25 s intervals. The stored values represented the root mean square (RMS) of the peak-to-peak acceleration amplitudes. Since acceleration is proportional to the rate of change of velocity and both quantities exhibited the same frequency characteristics, the peak-to-peak acceleration amplitude was chosen for further analysis as a sufficiently representative signal. Throughout the article, acceleration values are expressed in g (Earth’s gravitational acceleration).

**Figure 5:**
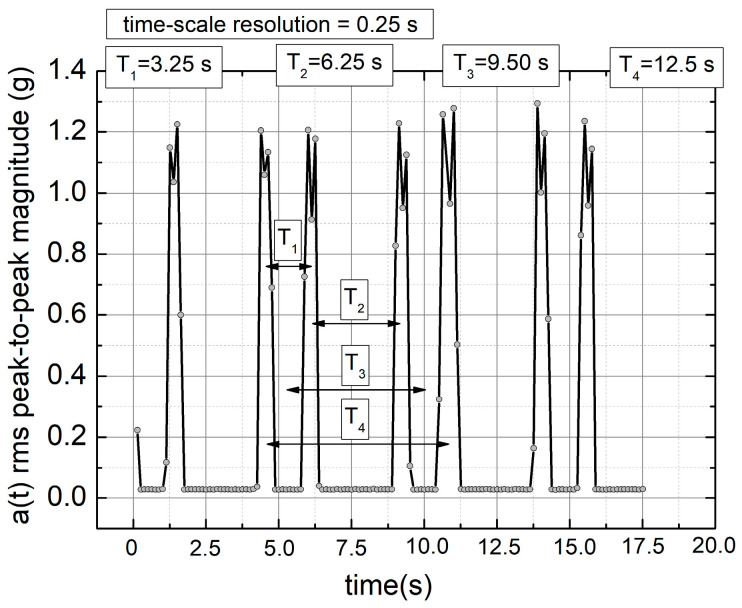
An example of the recorded acceleration RMS peak-to-peak magnitude values were recorded over time. The data revealed four distinct types of time periodicities. The figure highlights characteristic local time periods observed during the experiment. The collected acceleration signal, a(t), is expressed in units of g = 9.81 m/s^2^.

**Figure 6:**
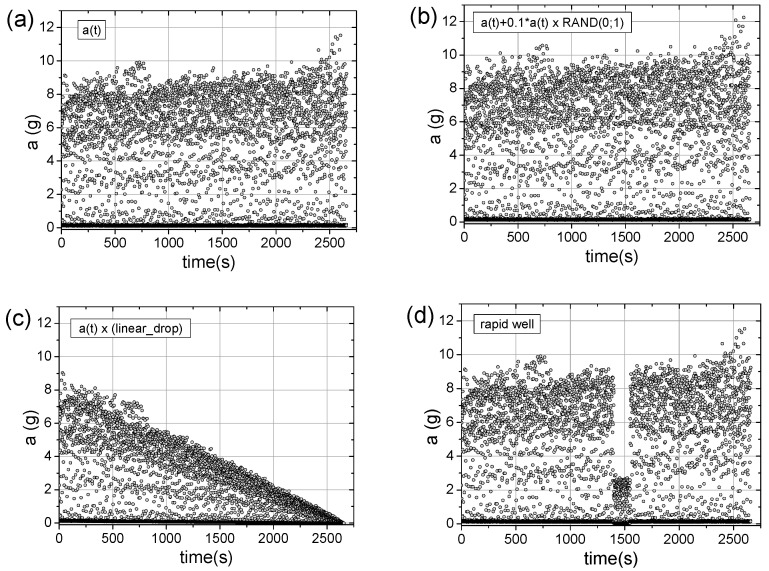
Time-dependent signals from the vibration sensor (averaged acceleration peak-to-peak values): (**a**) original signal, (**b**) signal with numerical modifications to mimic potential undesired disturbances, such as random instabilities, (**c**) signal with a linear decline, and (**d**) signal showing a sudden interruption (a “well”) in the working system. The collected acceleration signal, a(t), is expressed in units of g = 9.81 m/s^2^.

Figure 5 shows an example of several robotic work steps recorded by the acceleration sensor, highlighting characteristic time periods relevant to the TSM methodology. Figure 6a presents the data collected over 2700 s, while Figure 6b–d illustrates numerically introduced disturbances to the original signal. These cases are analyzed in detail below. Before proceeding, we introduce the TSM concept, which involves time-shifting a data series relative to the original to generate 2D representations.

### 3.1. Fast Fourier Transform (FFT) Analysis of Signals

**Figure 7:**
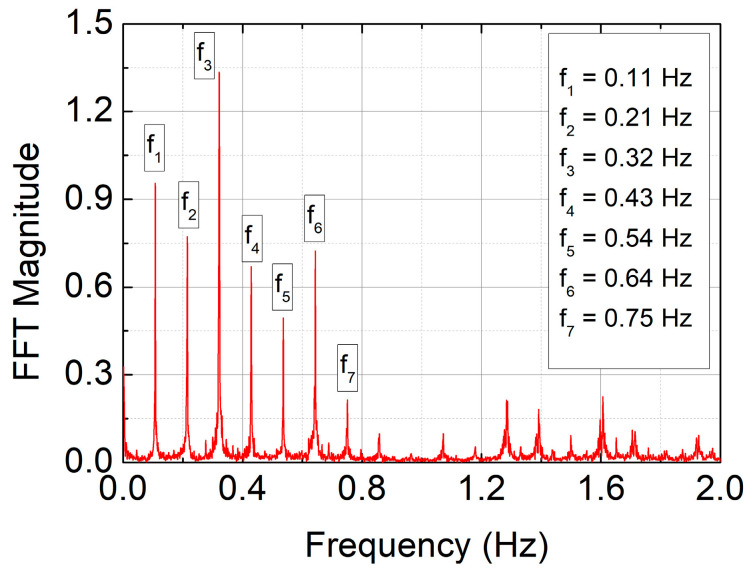
The FFT spectrum of the original signal (cf. Figure 6a). Characteristic frequencies related to the TSM method discussed in this study are marked in the figure.

The primary tool in industrial signal analysis may be the FFT method, especially for periodic signals, such as those discussed in this article. The analysis was conducted for a sample of 4096 measurement points, starting from time t = 1000 s (see Figure 6), specifically capturing the case of a sudden signal drop (see Figure 6d). The results of the FFT analysis are presented in Figure 7.

The FFT spectra obtained for all four types of signals analyzed in this study appear nearly identical, which is expected given the dominant periodic characteristics. The FFT method is sensitive to the frequency of periodic changes in a given parameter but does not indicate when specific features appear or disappear in time. For this reason, it is inherently unable to detect sudden events, such as the three-fold drop in signal amplitude shown in Figure 6d.

Nevertheless, it should be noted that the extracted signal frequencies, $f_{1}\ldots f_{7}$, as will be demonstrated later in this study, are related to the choice of time shift in the TSM method. Furthermore, to provide a broader perspective on the problem, we present below a wavelet analysis of the signals—a method capable of identifying not only the frequency components but also the specific moments when signal features appear or disappear.

### 3.2. Continuous Wavelet Transform (CWT) Analysis of Signals

**Figure 8:**
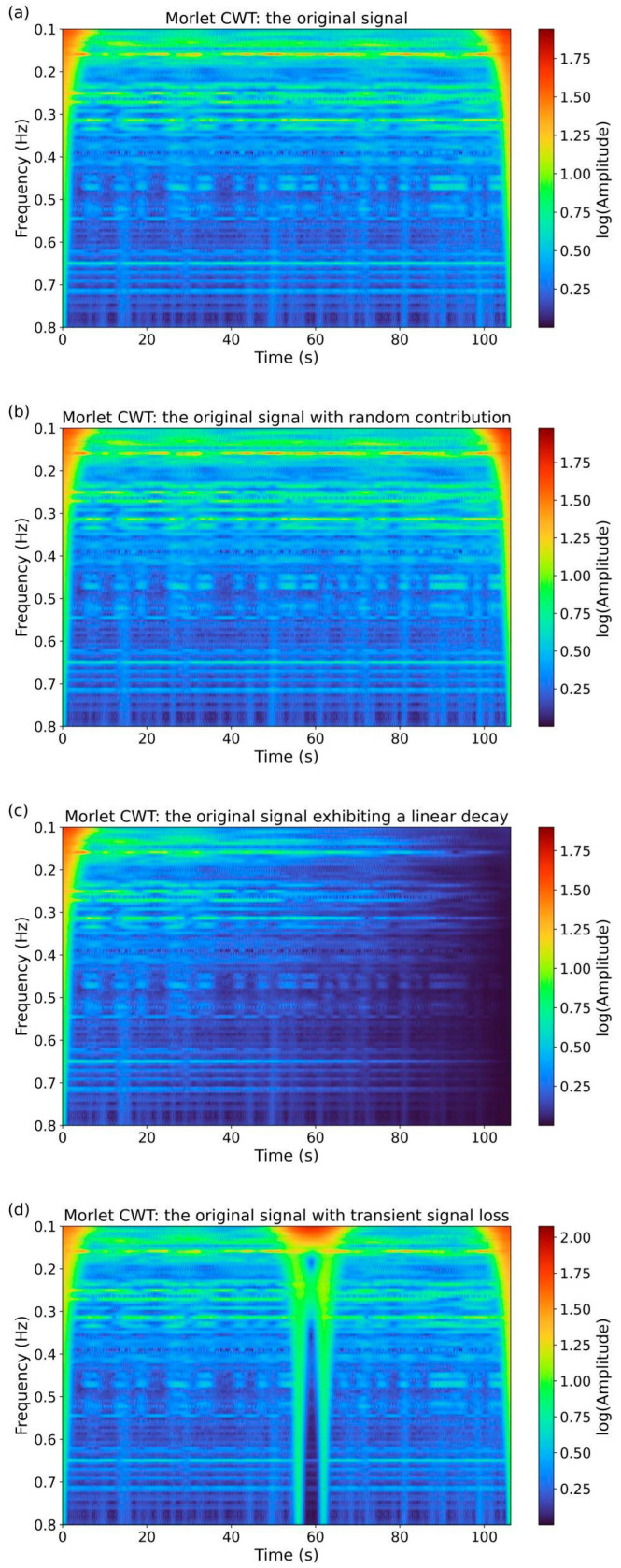
CWT analysis using the Morlet wavelet (bandwidth = 3.0, central frequency = 0.5). Spectrum of the analyzed signals: (**a**) the original signal; (**b**) the original signal with a random component; (**c**) the original signal exhibiting a linear decay to zero amplitude at the end of the recording; and (**d**) the original signal with a transient signal loss, where the amplitude was reduced three-fold between 1400 s and 1550 s. In all figures, the key frequency component of 0.11 Hz (identified as $f_{1}=0.11\;Hz$ Hz using FFT). Similarly, the FFT-identified $f_{2}=0.32\;Hz$ component is also distinctly present in the wavelet maps.

**Figure 9:**
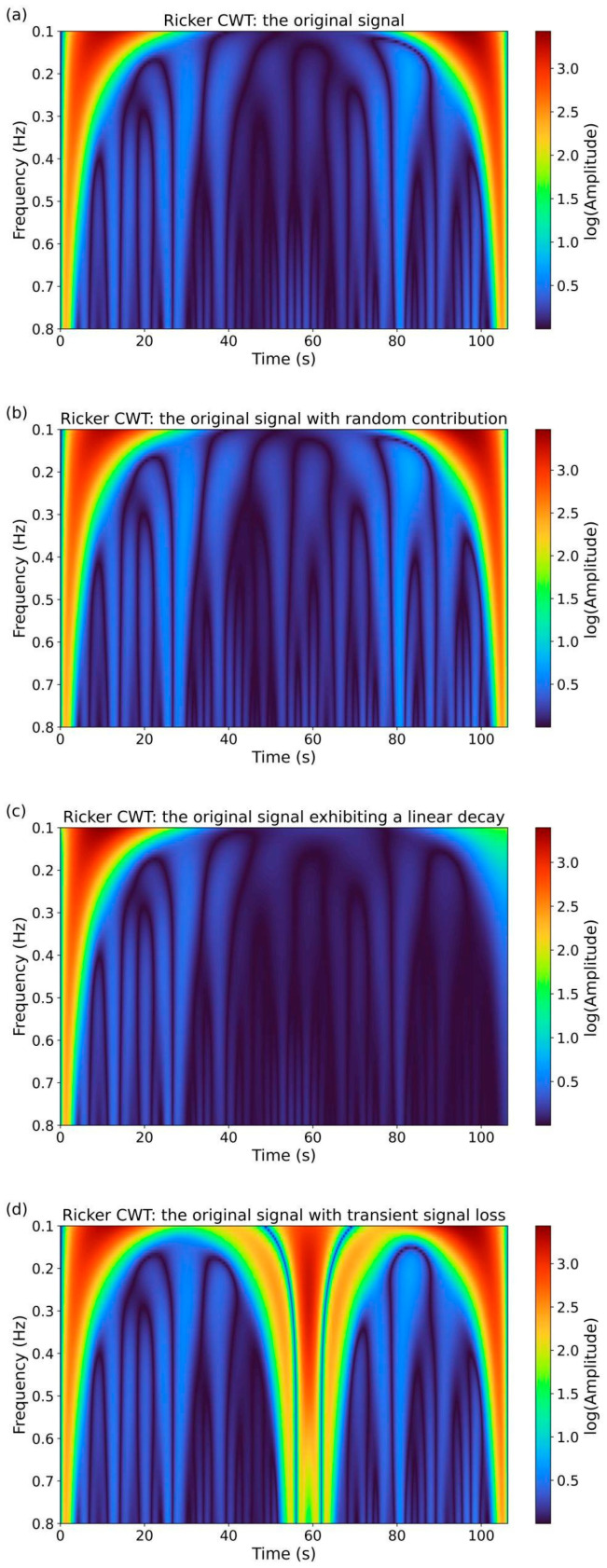
The CWT analysis using the Ricker (Mexican Hat) wavelet includes (**a**) the original signal; (**b**) the original signal with a random contribution; (**c**) the original signal exhibiting a linear decay to zero amplitude at the end of the recording period; and (**d**) the original signal with a transient signal loss, where the amplitude was reduced three-fold between 1400 s and 1550 s. The Ricker-based analysis provides good time-scale resolution, particularly in the case of transient signal loss.

Wavelet analysis was performed in Python (version 3.9.18) using the Spyder IDE (version 5.1.5). Two representative wavelets were employed: the Morlet wavelet (cmor3.0–0.5, bandwidth = 3.0, central frequency = 0.5, from the *pywt* package) and the Ricker wavelet (also known as the Mexican Hat), applied using the *cwt* and *ricker* functions from the *scipy.signal* package. In both cases, the sampling rate was set to 100 Hz. It is worth emphasizing that Morlet wavelets are well-suited for frequency analysis but are not intended for detecting transient phenomena in the time domain. Conversely, Ricker wavelets are effective for identifying aperiodic events in the time domain but do not offer detailed information about the frequency spectrum. Nevertheless, the result of wavelet analysis is always a two-dimensional map—representing the relationship between frequency and time. Figure 8 and Figure 9 present the wavelet analysis results for all four signals, with the usefulness of the Ricker wavelet becoming evident only in the case of the sudden signal drop (Figure 9c).

The obtained results, particularly those using the Morlet wavelet, provide a clear global overview of the situation, offering detailed insight into amplitude fluctuations of individual spectral components. Moreover, these results are consistent with the previously presented FFT findings. In general, the results obtained using the continuous wavelet transform method are not always clear and easy to interpret. As will be demonstrated in the following paragraph, the assessment of production process quality using the TSM approach appears to be more straightforward. Additionally, when supported by further calculations such as correlation dimension or image entropy, this approach yields an unambiguous numerical indicator that is easy to interpret and can enable a potentially quick response from production personnel. Wavelet analysis is not the primary focus of this study; interested readers are referred to the extensive literature on the subject, including [35,36,37,38,39].

### 3.3. Experimental Data Analysis Using TSM Approach

**Figure 10:**
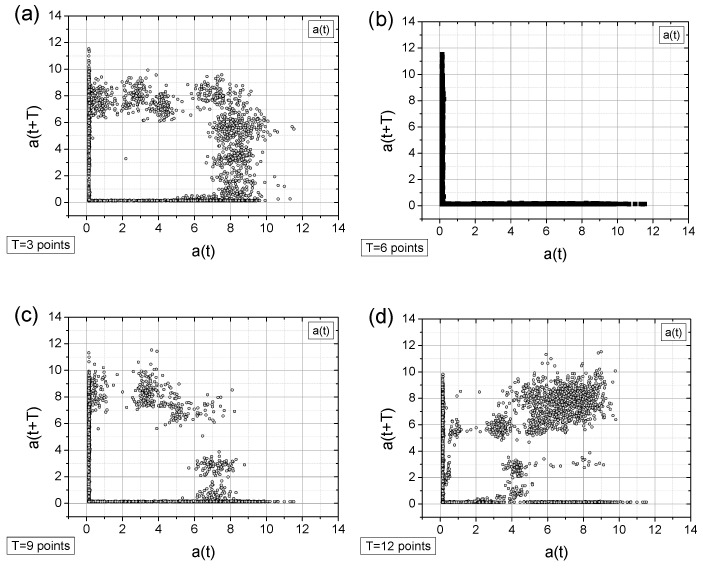

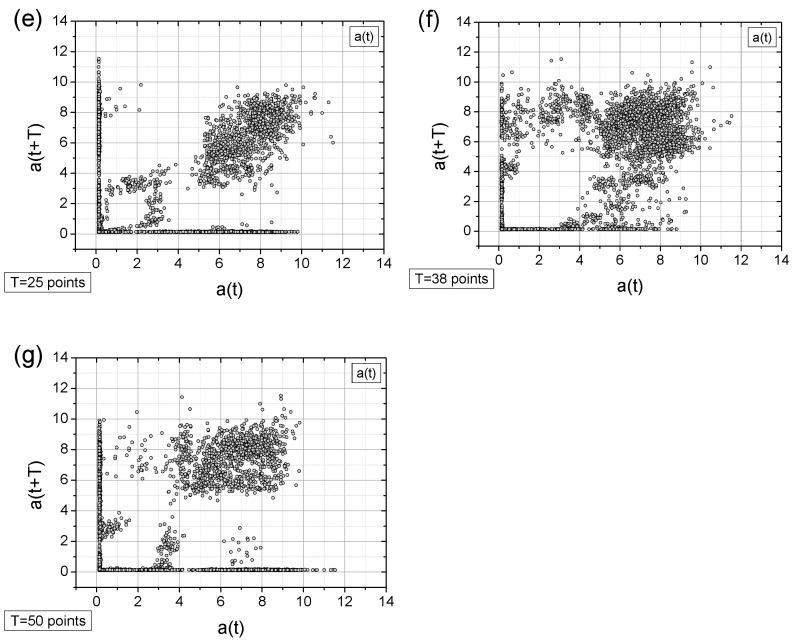
The TSM results for the originally collected acceleration signal. The acceleration signal, a(t), is expressed in units of g = 9.81 m/s^2^. The subfigures (**a**–**g**) differ in time shifts T values of which are given near the left-bottom corner.

**Figure 11:**
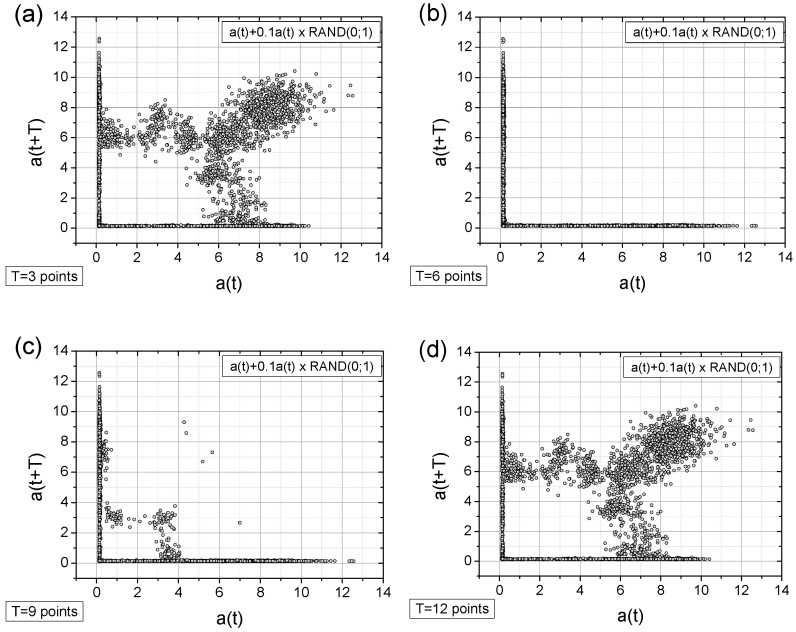

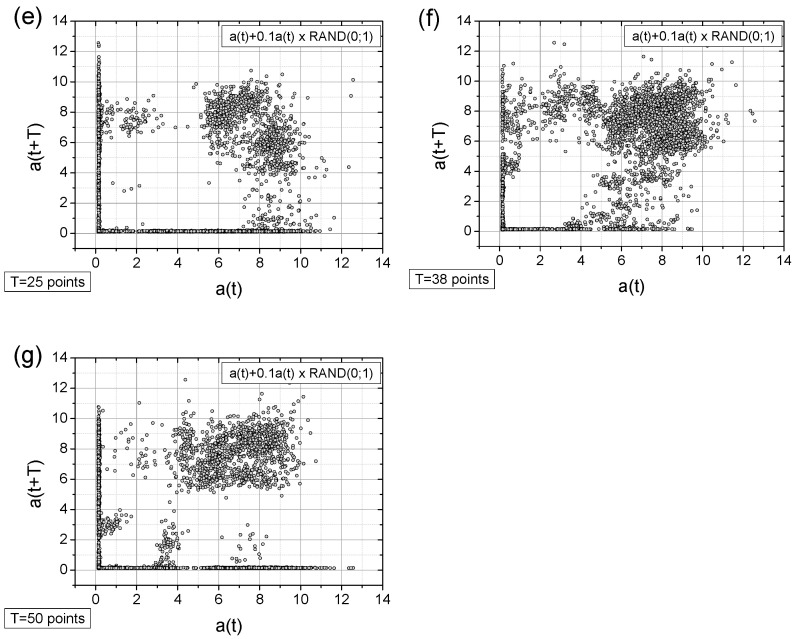
The TSM results for the originally collected acceleration signal with added random values (10% of the original values multiplied by a random value taken from the [0; 1] range)—cf. Figure 6b. The acceleration signal, a(t), is expressed in units of g = 9.81 m/s^2^. The subfigures (**a**–**g**) differ in time shifts T values of which are given near the left-bottom corner.

**Figure 12:**
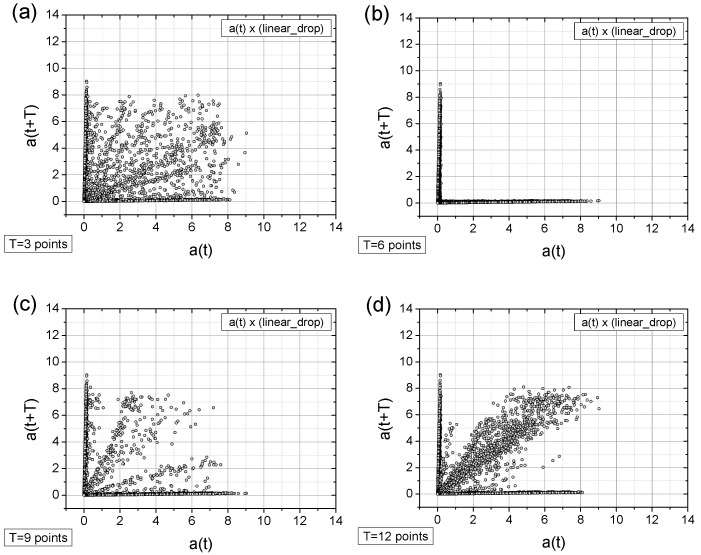

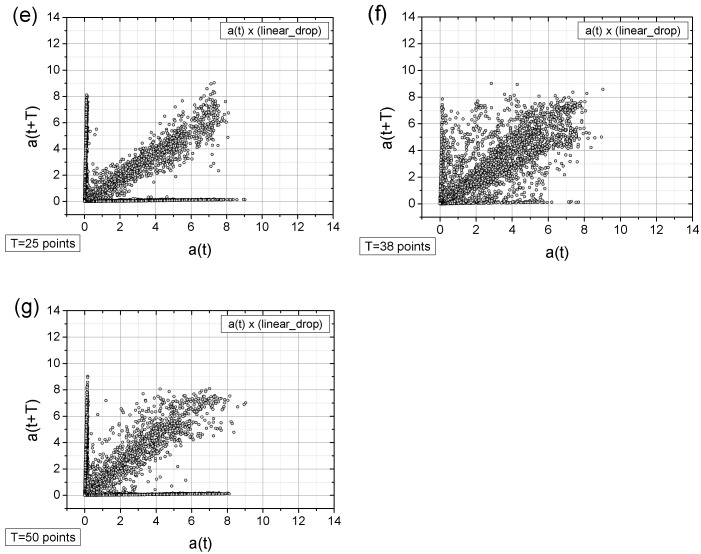
The TSM results for the originally collected acceleration signal with an imposed linear drop (cf. Figure 6c). The acceleration signal, a(t), is expressed in units of g = 9.81 m/s^2^. The subfigures (**a**–**g**) differ in time shifts T values of which are given near the left-bottom corner.

**Figure 13:**
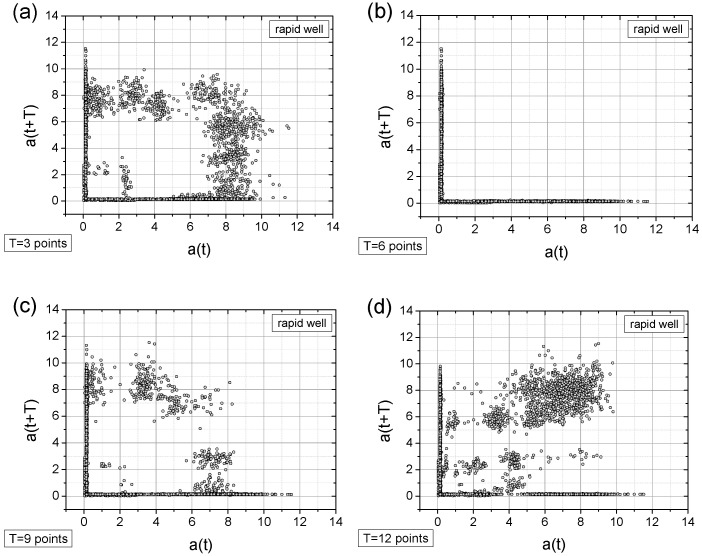

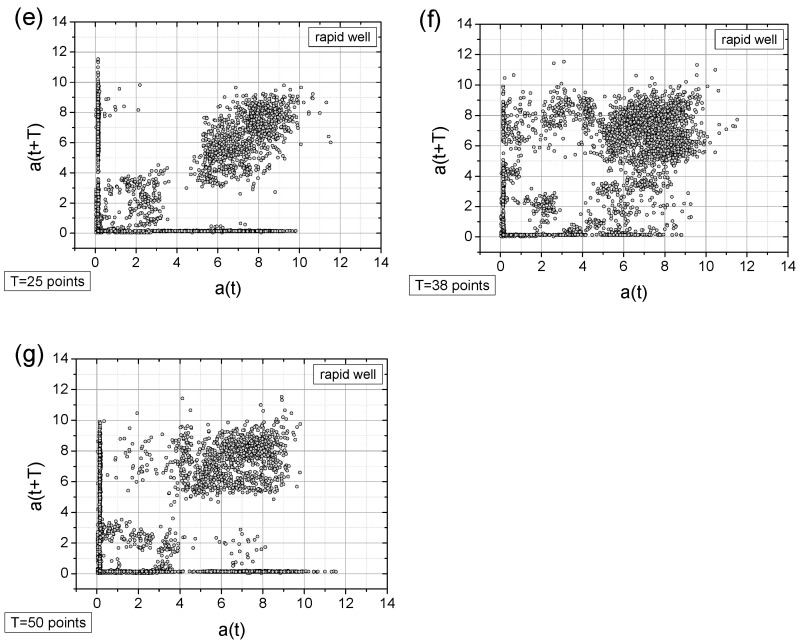
The TSM results for the originally collected acceleration signal with a sudden signal break (comp. Figure 6d). The acceleration signal, a(t), is expressed in units of g = 9.81 m/s^2^. The subfigures (**a**–**g**) differ in time shifts T values of which are given near the left-bottom corner.

Below, we present the TSM results for the originally registered data (Figure 10), data with artificially added noise (Figure 11), data with a linear signal drop lasting from the beginning to the end of the collection period (Figure 12), and data exhibiting a rapid drop (a “well”) in the signal (Figure 13). The maps were generated using different time shifts, expressed in “points” (time steps), where a single step corresponds to 0.25 s, as mentioned above. Since the periods shown in Figure 5 are equal to 3.25 s, 6.25 s, 9.5 s, and 12.5 s, the corresponding values of time shifts in the data points are 13, 25, 38, and 50, respectively. Similarly, considering the optimal choice of time shift as ¼ of the period, the respective values are 3, 6, 10, and 13 points. However, after evaluating various options, we present results below for time shift values (in points) of 3, 6, 9, 12, 25, 38, and 50, as these seem to be, at that moment, the most useful for characterizing the data.

As can be seen from the figures above, there is an unambiguous link between the current production state and the spatial distribution of measurement points. These distributions can be readily classified using appropriate analysis methods, like correlation dimension analysis or entropy calculations.

#### 3.3.1. Classification of TSM Results Using the Correlation Dimension Approach

The correlation dimension, also known as the frequency dimension, is particularly useful in situations where not only the spatial distribution of points within the phase space matters, but also where the local densities of points can be characterized [45]. The correlation dimension is based on the so-called correlation integral

(9) $$C\left(R\right)=\frac{1}{N(N-1)}\frac{1}{2}\sum_{i}^{N}\sum_{j}^{N}H\left(R-\left|r_{i}-r_{j}\right|\right),$$

where N denotes the number of experimental point pairs ($r_{i},r_{j}$) on the TSM map and H(x) is the Heaviside step function ($H\left(x\right)=1\;if\;x\geq 0,\;H\left(x\right)=0\;if\;x<0$), which serves as a counter for pairs of points located within a circle of radius R, centered at position $r_{i}$. The numerical procedure is repeated for various locations and different circle radii. In the present study, the typical size of the TSM bitmap was 3078 × 2740 pixels, after removing all unnecessary elements such as axes, grids, and captions. Each experimental point covered an area of 11 × 11 pixels. The correlation radius R ranged from 3 up to $\sqrt{{rows}^{2}+{columns}^{2}}$ with a step size of 1. For each selected radius, the counting experiment was repeated $\sqrt{rows\;x\;columns}$ times at random locations. The correlation dimension $d_{R}$ can be determined from the following relation

(10) $$C\left(R\right)\;\sim \;R^{d_{R}},$$

and can be derived from the slope of the following linear relationship

(11) $$log\left[C\left(R\right)\right]\;\sim \;d_{R}\log\left(R\right).$$

Hence, the simulations were performed using a custom MATLAB (version R2019b) script. The results are presented in Table 1. The numerical uncertainty associated with the estimated slope was never greater than 0.01.

**Figure 14:**
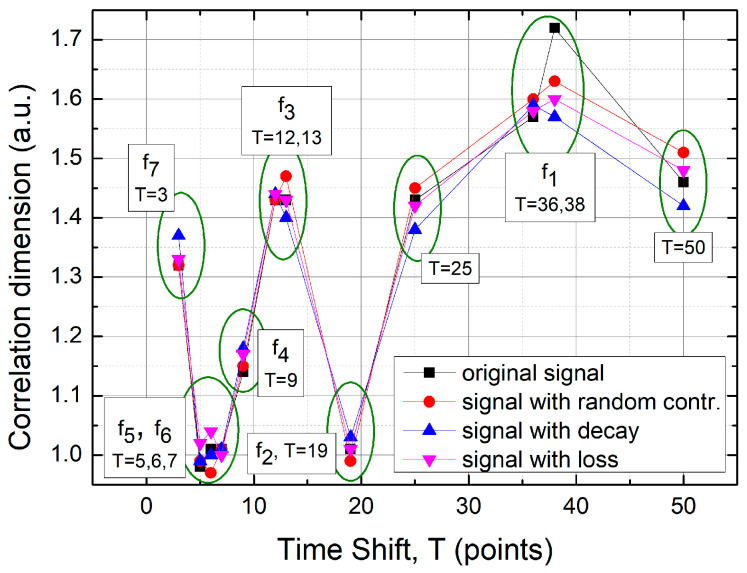
The correlation dimension as a function of time shift (T), along with its comparison to the FFT results. The best numerical resolution is obtained for T = 38, which corresponds to the FFT frequency $f_{1}=0.11\;Hz$.

The table above presents unique numerical values. The time shifts (T), shown in the first column, were obtained from the preliminary analysis—both through direct data extraction from Figure 4 and via the FFT method. The FFT clearly provides a valuable indication for selecting the appropriate time shift value. For instance, the frequency f_1_, corresponding to T = 36–37, offers the highest numerical resolution among the four analyzed cases in terms of correlation dimension. Nevertheless, shifts of 3 or 50 can also yield important graphical distinctions—see the TSM maps in the previous chapter (Figure 10, Figure 11, Figure 12 and Figure 13). All results are additionally summarized in Figure 14 below.

#### 3.3.2. Classification of TSM Results Based on Metric Entropy Calculations

Metric entropy, or missing information, is another quantity used to classify the distribution of experimental points on the TSM maps. It can be defined as:

(12) $$S=-\sum_{i=1}^{n}p_{i}log\left(p_{i}\right),$$

where it is assumed that the image is covered by a uniform grid of n^2^ boxes, and pᵢ is the probability of finding an experimental point within a given box. Here, we present results for a 50 × 50 box size. Table 2 provides the numerical results. The numerical uncertainty of the calculated entropies never exceeded 0.01.

**Figure 15:**
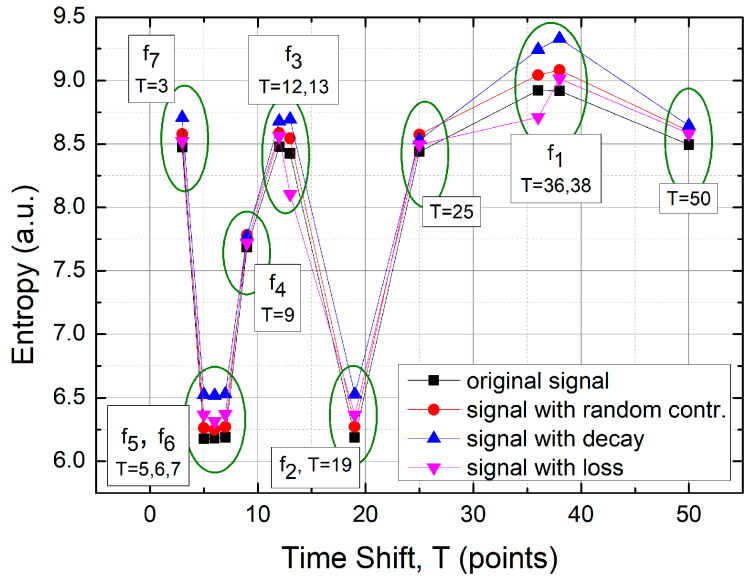
Metric entropy as a function of time shift (T) and its comparison with the FFT results. The highest numerical resolution is obtained for T = 36, the shift associated with the FFT frequency $f_{1}=0.11\;Hz$.

The entropy results offer even better numerical discrimination than the previous correlation dimension results, again for the $f_{1}$ frequency (T = 36). This improved recognition is also evident for all other frequencies, especially for $f_{3}$ (T = 13), where the rapid transient signal loss is clearly detectable. Figure 15 summarizes these results.

## 4. Discussion and Conclusions

A closer look at the obtained results reveals the following observations:

- The original signal exhibits a single, approximately dominant type of periodicity (one loop), which is particularly evident in the maps with a time shift of T = 3 points (Figure 10a, Figure 11a and Figure 13a) and less apparent in the linearly descending signal (Figure 12a).
- In general, all charts, except those with a time shift of T = 6 points, proved to be effective for analyzing the registered signals.
- Adding a random factor to the signal disrupts its periodicity: On a map with a time shift of T = 9 points (cf. Figure 11a,c), the map completely changes its character.
- The linear decay of the signal results in the appearance of new collinear sets of points on the chart (e.g., Figure 12c).
- Rapid signal decay (a well) leads to the appearance of a signal (one loop) with a smaller amplitude (cf. Figure 10a and Figure 13a)—indicating the two-frequency case. In Figure 13a, there is a small quadratic quasi-loop approximately 2 × 2 in size.
- Along with the observed periodicities, areas of high randomness are clearly visible—cf. maps for T = 25 points, particularly for accelerations exceeding 5 g (depending on the signal type).
- The choice of time shift (T) can be supported by the FFT method and effectively classified using the computed metric entropy.

The graphical data presentation method proposed in this paper, the approach related to deterministic chaos methods, demonstrates sensitivity to the nature of the collected data, effectively distinguishing between two dominant components: periodic and stochastic. By applying various modifications to the original signals, the TSM method has proven to be both representative and selective for the cases presented in this analysis.

In conclusion, the introduced time-shifted maps (TSMs) represent a novel approach to industrial data analysis, offering significant advantages over traditional methods in detecting hidden patterns and abnormal situations in time–domain signals. TSM, based on a simple algorithm, not only generates clear visual representations but also enables effective data classification using computed correlation dimensions and metric entropy measures. A comparison of the results obtained using this method with those from classical signal processing techniques, such as the discrete Fourier transform (FFT) and wavelet transform, demonstrates TSM’s superiority in identifying subtle yet critical anomalies affecting process stability. Grounded in straightforward mathematical principles, the method proves to be a valuable tool for supporting production process control and monitoring the technical condition of industrial automation systems.

Furthermore, the resulting maps, which reflect modifications caused by changes in technological parameters, are highly suitable for precise and unambiguous analysis, such as employing unsupervised machine learning clustering techniques. This area of application remains underexplored and will be the focus of future research on industrial data obtained from operational robotic systems.

## Figures and Tables

**Table 1:** The results of the correlation dimension were calculated for the following four cases: the original signal, the original signal with a random contribution, the original signal exhibiting linear decay, and the original signal with transient signal loss (a rapid drop).

Time Shift T (Points)	Original Signal	Signal with Random Contr.	Signal with Decay	Signal with Loss
3	1.32	1.32	1.37	1.33
5	0.98	0.99	0.99	1.02
6	1.01	0.97	1.00	1.04
7	1.01	1.01	1.01	1.00
9	1.14	1.15	1.18	1.17
12	1.43	1.43	1.44	1.44
13	1.43	1.47	1.40	1.43
19	1.01	0.99	1.03	1.01
25	1.43	1.45	1.38	1.42
36	1.57	1.60	1.59	1.58
38	1.72	1.63	1.57	1.60
50	1.46	1.51	1.42	1.48

**Table 2:** Results of metric entropy calculated for the four cases of: the original signal, the original signal with a random contribution, the original signal exhibiting linear decay, and the original signal with transient signal loss (rapid well).

Time Shift T (Points)	Original Signal	Signal with Random Contr.	Signal with Decay	Signal with Loss
3	8.47	8.58	8.71	8.52
5	6.18	6.26	6.52	6.37
6	6.18	6.25	6.52	6.32
7	6.19	6.27	6.53	6.37
9	7.69	7.78	7.77	7.72
12	8.48	8.59	8.68	8.56
13	8.43	8.54	8.69	8.10
19	6.19	6.27	6.53	6.37
25	8.44	8.57	8.53	8.50
36	8.92	9.04	9.24	8.71
38	8.92	9.08	9.33	9.01
50	8.50	8.60	8.64	8.58

## Data Availability

The original contributions presented in the study are included in the article, further inquiries can be directed at the corresponding author.
